# Marginal Integrity of Simplified Adhesive Strategies in Primary Teeth

**DOI:** 10.1016/j.identj.2023.06.002

**Published:** 2023-06-28

**Authors:** Mia de Boer, Marcus Zimmermann, Thomas Attin, Tobias T. Tauböck, Blend Hamza

**Affiliations:** a Clinic of Conservative and Preventive Dentistry, Center of Dental Medicine, University of Zurich, Zurich, Switzerland; b Clinic of Orthodontics and Pediatric Dentistry, Center of Dental Medicine, University of Zurich, Zurich, Switzerland

**Keywords:** Selective enamel etch, Primary teeth, Self-etch, Shortened application time, Universal adhesives

## Abstract

**Objective:**

The aim of this research was to investigate the effect of simplified adhesive strategies (self-etch vs selective enamel etch and 10- vs 20-second adhesive application time) on the marginal integrity in primary molars.

**Methods:**

Forty deep class-II cavities were prepared in 40 extracted primary molars. The molars were divided into 4 groups based on the applied universal adhesive strategy as follows: groups 1 and 2: selective enamel etch with 20- or 10-second application time and groups 3 and 4: self-etch with 20- or 10-second application time. All cavities were restored with a sculptable bulk-fill composite restoration. The restorations underwent a thermomechanical loading (TML, 5–50 °C, 2-minute dwelling time, ×1000; 400,000 loading cycles, 1.7 Hz, 49 N). Marginal analysis before and after TML was conducted with scanning electron microscopy and the marginal integrity of each restoration was calculated as a percentage of continuous margins. A beta regression model was adopted to statistically analyse the data with a consequent pairwise comparison.

**Results:**

The mean marginal integrity (% ± SD) of the restorations for each tested adhesive strategy after TML was as follows: selective enamel etch/20 seconds = 85.4 ± 3.9, self-etch/20 seconds = 85.3 ± 5.2, self-etch/10 seconds = 80.1 ± 8.2, and selective enamel etch/10 seconds = 80.0 ± 8.5. The difference between both adhesive strategies was not statistically significant at the same application time. The difference between both application times within the same adhesive strategy was statistically significant (*P* ≤ .01).

**Conclusions:**

Universal adhesives applied either in selective enamel etch or in self-etch mode result in comparable marginal integrities when restoring class-II cavities in primary molars. Shortened adhesive application time (10 seconds) could lead to a reduction in the marginal integrity in comparison to the recommended application time of 20 seconds.

## Introduction

Placing direct restorations is one of the most frequent procedures carried out by dentists.^1^ Amongst those restorations, resin-based composites are commonly used restorative materials. Traditionally, 3-step bonding agents were used to make the composite adhere to enamel and dentine. These steps include etching with 37% phosphoric acid, rinsing and drying, applying a primer containing hydrophilic monomers (HEMA), and using an adhesive to form the interface between hydrophilic dentine and hydrophobic composite. These steps were, however, deemed as technique-sensitive and time-consuming, and the demand for simpler adhesives remained high.^2^ A simplification of the adhesion procedure was introduced by developing universal adhesives. Universal adhesives can be applied either in self-etch mode (without a prior etch-and-rinse step) or in combination with a prior total (enamel and dentine) or selective enamel etch-and-rinse step.^3^ Chen et al stated that there was no statistically significant difference between universal adhesives applied in self-etch or etch-and-rinse mode regarding immediate and long-term bond strength on dentine.^4^ Applying universal adhesives in self-etch mode relies on the simultaneous etching and infiltration of enamel and dentine due to the presence of acidic functional monomers (10-methacryloyloxydecyl dihydrogen phosphate [10-MDP]).^5^ Manufacturers usually recommend a specific application time for their universal adhesives to be rubbed on the cavity walls. However, as a further attempt to simplify the restoration procedure, some studies have investigated the effect of shortening the recommended application time on the performance of these adhesives. These studies revealed that the shortened application time compromises the performance of universal adhesives.^6^^,^^7^

Primary enamel is generally thinner and less mineralised than enamel of permanent teeth.^8^ Accordingly, enamel of primary teeth has greater susceptibility to demineralise in acidic media.^9^ Furthermore, the density of dentinal tubules was observed to be higher in primary teeth compared to permanent ones.^10^ Whilst primary dentinal tubules show a straight course, the dentinal tubules of permanent teeth follow an “S”-shaped curve.^11^ With these differences in mind, the fact that adhesives seem to be more effective in permanent teeth than in primary ones, in terms of bond strength and preventing gingival-wall microleakage in class-II composite restorations, could be partially explained.^10^

All the above-mentioned strategies that aim to shorten the restoration procedure could be beneficial in paediatric dentistry as increased treatment durations when treating children was reported to have a negative effect on their cooperation.^12^ However, the application of such simplifying strategies (omitting the etch-and-rinse step and reducing the adhesive application time) on primary teeth has not yet been investigated. This study was therefore carried out to investigate the effect of simplified adhesive strategies (self-etch vs selective enamel etch and 10- vs 20-second adhesive application time) on the marginal integrity of a bulk-fill composite in class-II cavities in primary molars.

## Methods

Forty extracted primary molars with a single sound proximal surface were included in this in vitro study. The extraction was indicated due to pulp necrosis (furcation radiolucency and/or the formation of a fistula or an extraoral abscess) or for orthodontic reasons. The molars were anonymised and stored in 0.5% Chloramine-T solution at 4 °C until they were used. Written consent was signed by the parents and children, and the study was carried out in agreement with the Federal Act on Research involving Human Beings (Human Research Act; article 2, paragraph 2). The authorisation from the ethics committee was waived from the ethics commission (Zurich cantonal ethics commission, BASEC-2022-00961).

**Fig. 1:**
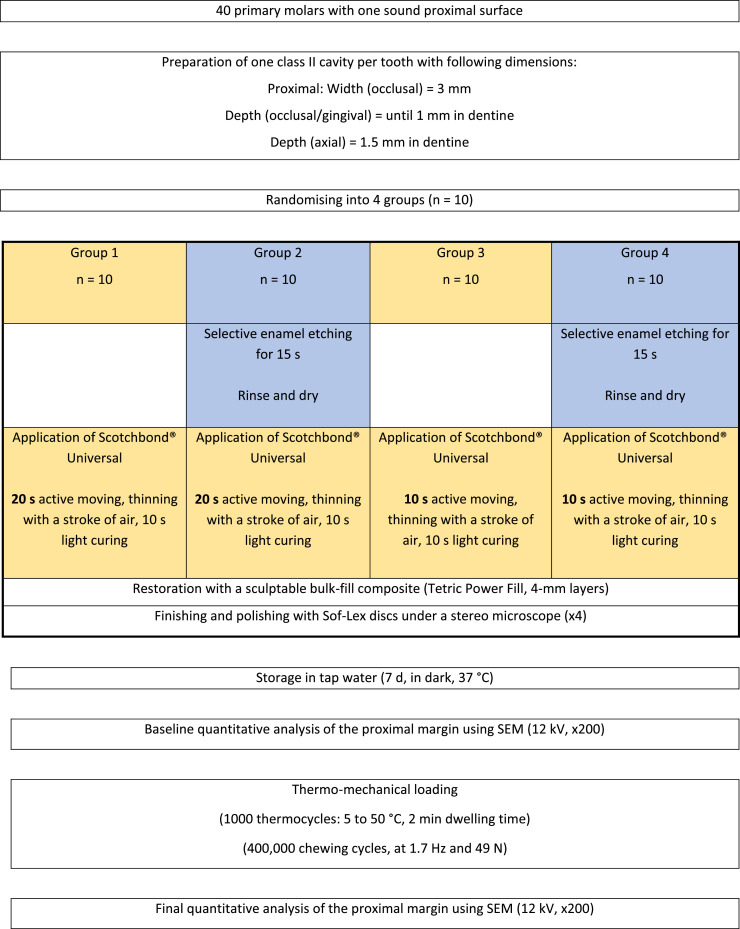
Study design.

The molars were embedded in acrylic resin (Paladur, Heraeus Kulzer, Hanau, Germany) 3 mm below the cemento-enamel junction. Using an 80-µm cylindrical bur, standardised proximal cavities were prepared on the sound proximal surface of each molar (slot preparation). The cavities’ dimensions are shown in Figure 1. Each molar was then assigned to a code (engraved on the acrylic embedding material) and randomised into 4 groups (n = 10) based on the applied adhesive strategy and using a computer-generated randomisation table (Microsoft Excel, Microsoft). No statistical sample size calculation was carried out. Earlier marginal integrity studies were used as guidance for this matter.^13^, ^14^, ^15^, ^16^, ^17^

Molars were fixed on a custom-made adjacent-tooth simulator. A stainless-steel matrix band was placed and fixed with a wooden wedge. In group 1, a universal adhesive (Scotchbond Universal, 3M) was applied on the cavity walls for 20 seconds. The same procedure was applied in group 2 after performing a selective enamel etch (15 seconds) and a rinse-and-dry step. In group 3, the same universal adhesive was applied on the cavity walls for 10 seconds. The same procedure was applied in group 4 after performing a selective enamel etch (15 seconds) and a rinse-and-dry step. The adhesive was thinned with a gentle blow of oil-free air and light cured for 10 seconds at 1340 mW/cm^2^ (Bluephase PowerCure, Ivoclar Vivadent). All cavities were then restored with a sculptable bulk-fill composite placed in 4-mm layers (Tetric Power Fill, Ivoclar Vivadent). Each composite layer was light cured for 10 seconds at 1340 mW/cm^2^. The restorations were wet-polished using decreasing grit-size Sof-Lex discs (3M ESPE) under a stereo microscope (×4). The restored molars were kept in tap water inside a dark incubator (37 °C) for 7 days.

**Fig. 2:**
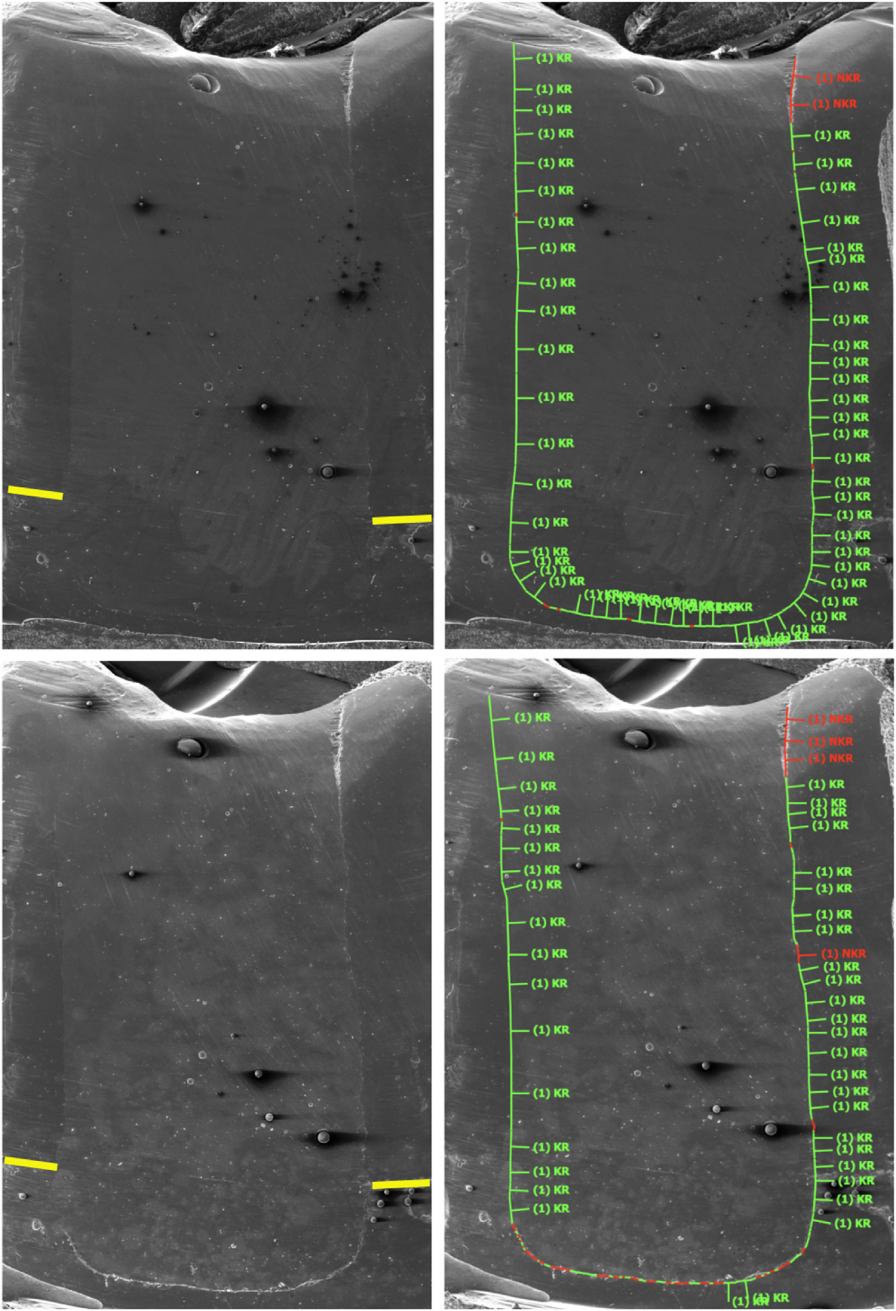
Scanning electron microscopy images (×200) and the quantitative marginal analysis (right) for a restoration before (upper images) and after thermomechanical loading (lower images). The green lines indicate continuous margin segments, and the red lines indicate noncontinuous margin segments. The yellow line indicates the cemento-enamel junction.

Impressions of the restorations were taken using A-silicon material (President Light Body, Coltene Whaledent). The impressions were poured out (Epoxyharz L, R&G Faserverbundwerkstoffe) and fixed on aluminium holders (Cementit Universal, Merz & Benteli), and the formed replicas were sputter-coated with gold (Sputter SCD 030, Balzers Union, Balzers, Liechtenstein). The initial marginal integrity of the restorations was quantitatively analysed using scanning electron microscopy (SEM; 20 kV, 200× magnification) (Amray 1810/T, Amray) and was expressed as a percentage of the assessable continuous margins of the respective restoration using a custom-made programme (4 Dimensions, ZPZ). Afterwards, thermo-mechanical loading (TML) was applied on each restoration inside an electronic masticator (CoCoM 2, ZPZ). Three-millimetre metal balls were used as antagonists to chew over the restorations (400,000 chewing cycles at 49 N), and the water temperature was changed from 5 to 50 °C (1000 thermocycles). After TML, new A-silicon impressions were taken for the restorations and final marginal integrity analysis was performed using the same protocol. Marginal analysis was conducted by one calibrated operator (MZ) who was blinded to the groups and had access only to the SEM images (file name = code set for each molar). Figure 1 summarises the study design and Figure 2 depicts the marginal analysis of one restoration.

### Statistical analysis

The marginal integrity was set as the target variable and was investigated with respect to the explanatory variables (adhesive strategy and application time). Due to the data character, beta regressions were used for data modelling.^18^ Observations at 10-s frequently showed higher variance in the target variable than observations at 20-s. This data behaviour was accounted for by explicitly estimating the variance as well (non-constant variance). The beta regression modelling with non-constant variance was implemented using the gamlss package in R.^19^ The post hoc pairwise comparisons between the contrasts were then computed using the emmeans package. The significance level was set at *p* ≤ 0.05.

## Results

### Marginal integrity of the whole restoration

Before TML, the mean ± standard deviation (SD) of each experimental group was calculated. The achieved marginal integrity (%) for each combination was as follows: selective enamel etch/20 seconds = 94.5 ± 2.5, self-etch/20 seconds = 92.7 ± 4.2, selective enamel etch/10 seconds = 89.3 ± 6.4, and self-etch/10 seconds = 87.2 ± 7.5.

**Fig. 3:**
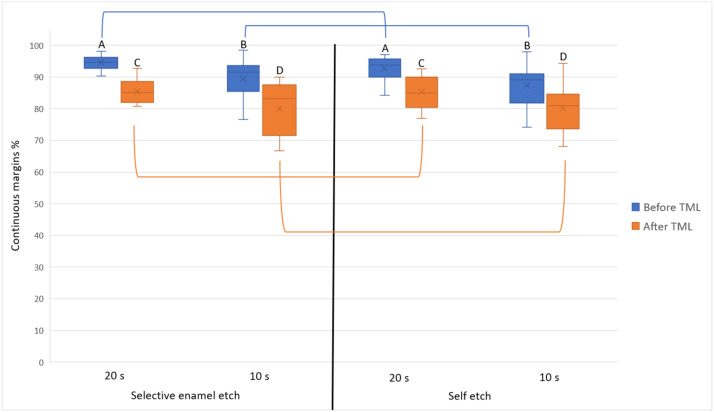
Marginal integrity of the whole restoration (means = x marks, medians: horizontal lines, quartiles = boxes, interquartile range = whiskers). Same letters indicate no statistically significant difference between different application times within the same adhesive strategy. Connecting lines indicate no statistically significant difference between the adhesive strategies within the same application time.

After TML, the achieved marginal integrity for each combination was as follows: selective enamel etch/20 seconds = 85.4 ± 3.9, self-etch/20 seconds = 85.3 ± 5.2, self-etch/10 seconds = 80.1 ± 8.2, and selective enamel etch/10 seconds = 80.0 ± 8.5. The difference between both adhesive strategies was not statistically significantly different at the same application time (*P* ≥ .5). The difference between both application times within the same adhesive strategy was, however, statistically significant (*P* ≤ .01). Figure 3 depicts the achieved marginal integrity of the whole restoration.

### Marginal integrity only within enamel

Before TML, the achieved marginal integrity (% ± SD) of the restorations’ parts within enamel for each combination was as follows: selective enamel etch/20 seconds = 95.0 ± 3.2, self-etch/20 seconds = 92.3 ± 5.4, selective enamel etch/10 seconds = 91.0 ± 8.1, and self-etch/10 seconds = 84.5 ± 9.5.

**Fig. 4:**
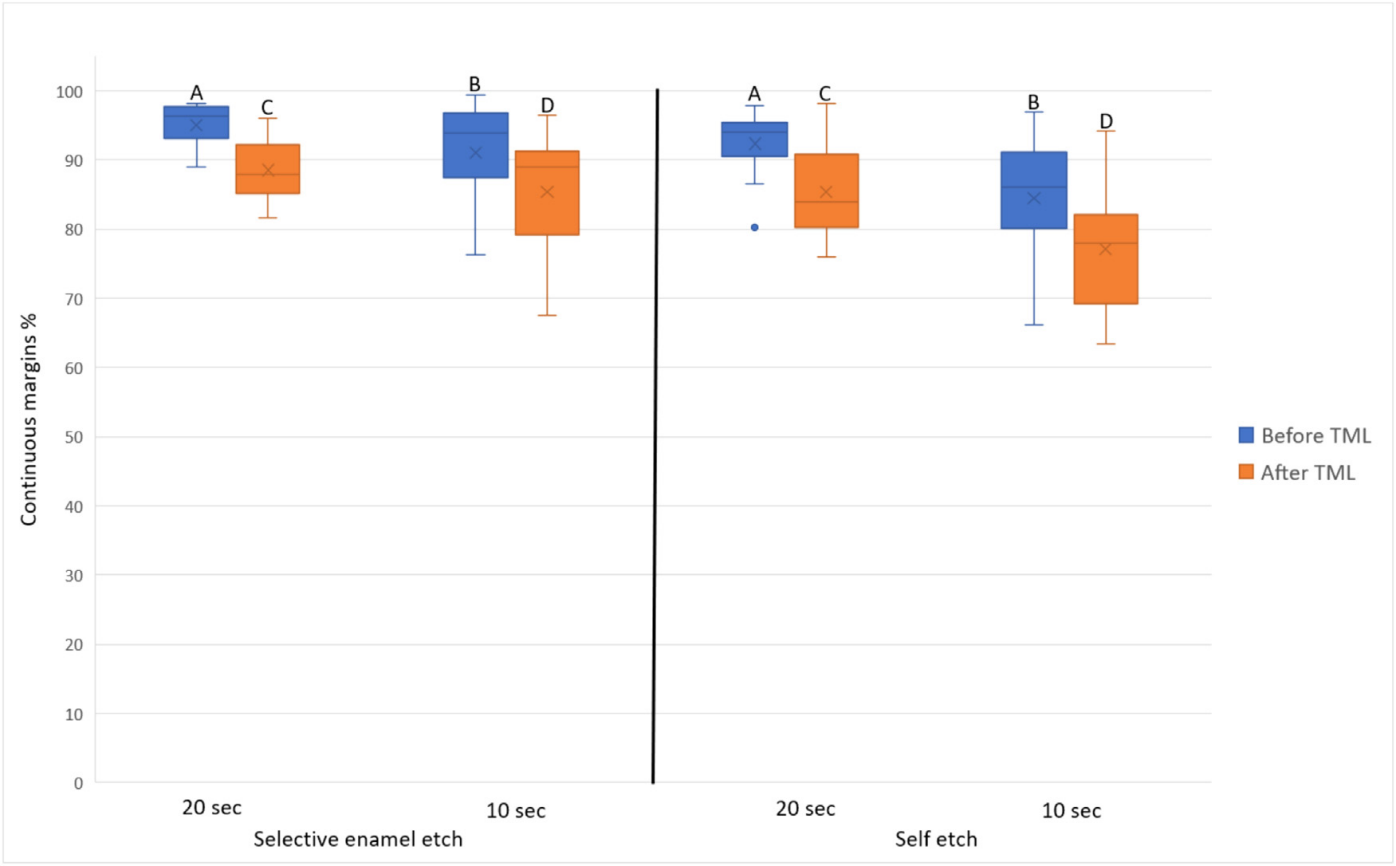
Marginal integrity within enamel (means = x marks, medians: horizontal lines, quartiles = boxes, interquartile range = whiskers). Same letters indicate no statistically significant difference between different application times within the same adhesive strategy. Difference between the adhesive strategies within the same application time was always statistically significant.

After TML, the achieved marginal integrity for each combination was as follows: selective enamel etch/20 seconds = 88.5 ± 5.2, self-etch/20 seconds = 85.4 ± 7.3, selective enamel etch/10 seconds = 85.4 ± 9.1, and self-etch/10 seconds = 77.1 ± 10.0. The difference between both adhesive strategies was statistically significant at the same application time (*P* = .05). The difference between both application times within the same adhesive strategy was also statistically significant (*P* ≤ .05). Figure 4 depicts the achieved marginal integrity of the restorations only within enamel.

### Marginal integrity only within dentine

Before TML, the achieved marginal integrity (% ± SD) of the restorations’ parts within dentine for each combination was as follows: selective enamel etch/20 seconds = 93.5 ± 3.1, self-etch/20 seconds = 93.3 ± 5.6, self-etch/10 seconds = 91.6 ± 6.8, and selective enamel etch/10 seconds = 85.5 ± 5.4.

**Fig. 5:**
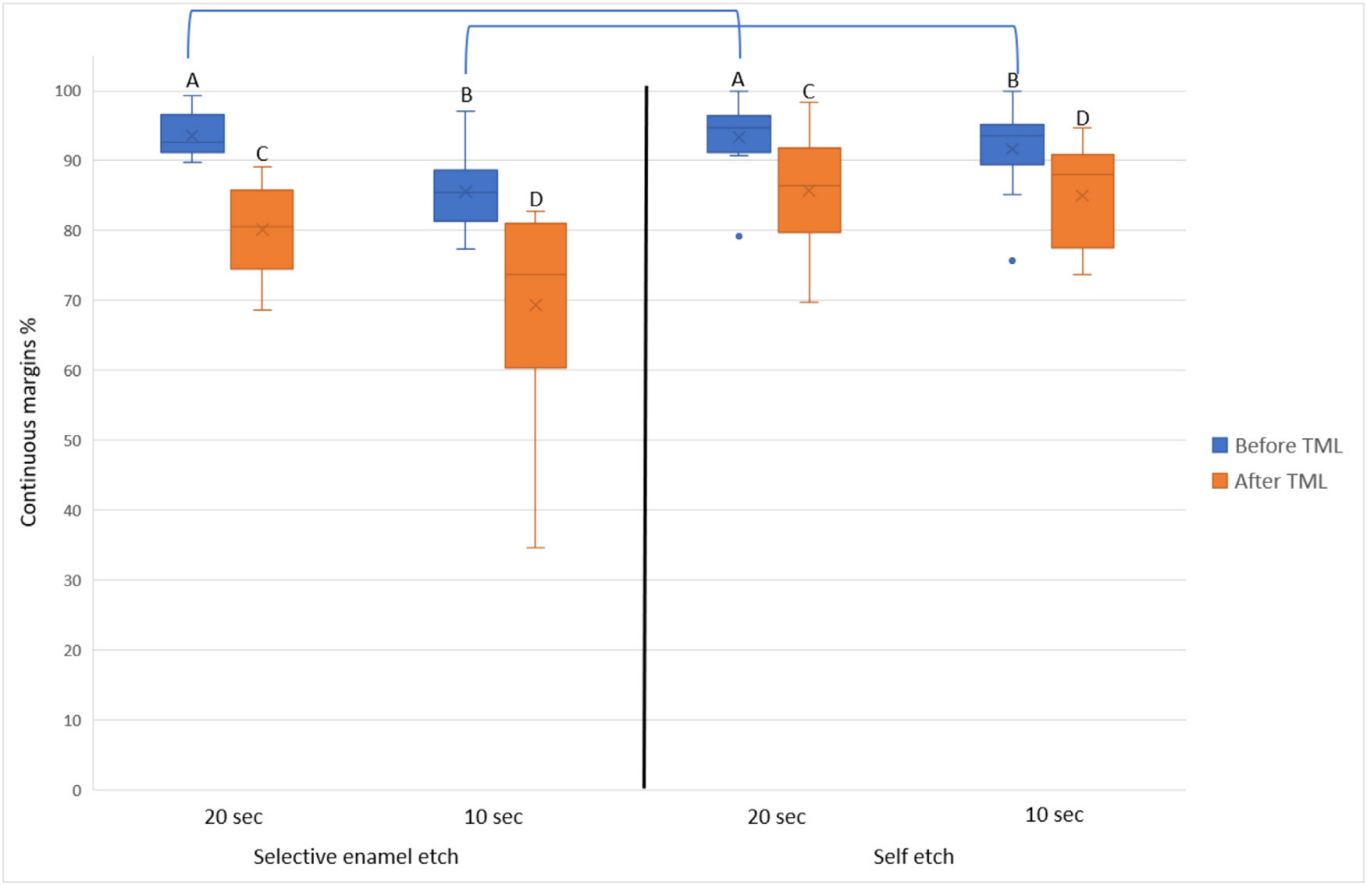
Marginal integrity within dentine (means = x marks, medians: horizontal lines, quartiles = boxes, interquartile range = whiskers). Same letters indicate no statistically significant difference between different application times within the same adhesive strategy. Connecting lines indicate no statistically significant difference between the adhesive strategies within the same application time.

After TML, the achieved marginal integrity for each combination was as follows: self-etch/20 seconds = 85.6 ± 8.1, self-etch/10 seconds = 85.0 ± 7.5, selective enamel etch/20 seconds = 80.1 ± 6.6, and selective enamel etch/10 seconds = 69.3 ± 15.2. The difference between both adhesive strategies was statistically significant at the same application time (*P* ≤ .001). The difference between both application times within the same adhesive strategy was also statistically significant (*P* ≤ .05). Figure 5 depicts the achieved marginal integrity of the restorations only within dentine.

## Discussion

Reducing steps and time to perform restorations in primary teeth could be beneficial in paediatric dentistry. This study investigated the marginal integrity of a bulk-fill restoration when using simplified adhesive strategies, namely when applying a universal adhesive in self-etch mode (vs selective enamel etch mode) and in a shortened application time (10 vs 20 seconds). To the authors’ knowledge, this is the first in vitro study to compare these adhesive strategies in primary teeth.

The cervical margins of the cavity ended in dentine in this study. Taking the crown convergence of primary molars into consideration, it could be argued that this dimension is rather deep and could clinically lead to restoration difficulties (when placing a matrix band).^20^^,^^21^ At the same time, restoring such deep cavities with the tested adhesive strategies enables a solid evaluation of their performance. All cavities were restored with a sculptable bulk-fill composite material. Two recent studies already reported sculptable bulk-fill composites (inserted in 4-mm layers) to perform similarly as classical composites in terms of marginal integrity in primary molars.^13^^,^^22^ The applied chewing load (49 N) and frequency (1.7 Hz) are reported most often in the literature for composite restorations.^23^ According to ISO (11405), 500 thermocycles would represent a suitable short-term aging protocol of dental materials.^24^ In any case, there is a lack in the literature regarding the correspondence between the TML cycles and the clinical service time of a composite restoration.^23^ Although the TML used here attempted to mimic the in vivo situation of chewing and consuming hot/cold beverages, other environmental factors were not mimicked in this study (eg, changes in saliva pH level, influence of abrasion due to tooth brushing). The assessment of the marginal integrity in this study was carried out using the indirect impression method. Although this procedure helps assess the margins at different stages through the study (before and after TML),^25^ it holds certain drawbacks. These include possible interaction between the impression material and parts of the adhesive system that might interfere with the impression accuracy as well as lack of an exact protocol of impression taking.^26^ Another limitation of this study is the fact that it only considered the marginal integrity of the restorations. Other factors (bond strength, microleakage) should also be considered when investigating a restoration or an adhesive strategy.^25^ Furthermore, it has not been proven that marginal gaps correlate with the development of marginal secondary caries.^26^ Future studies could investigate the effect of simplified adhesive strategies on the bond strength and the whole clinical success of the restorations as well as on the cooperation of treated children.

With the high success rates of the Hall technique (placing a stainless-steel crown on a carious vital asymptomatic primary molar without any caries excavation or preparation),^27^ the logic behind further investigations regarding composite restorative procedures in primary teeth might be in question. However, this technique is still not considered the “gold standard” by numerous general and paediatric dentists.^28^ Furthermore, only a small proportion of dentists familiar with and using the Hall technique reported that they would use it over conventional restorative methods in a case deemed ideal for the Hall technique.^28^ When considering the enamel part of the restorations (Figure 4), selective enamel etch always offered a statistically significantly better marginal integrity than self-etch. However, this was not the case when considering the whole restoration (Figure 3). It could be argued here that the dentine parts of the restoration had lower marginal integrity than the enamel parts, which led to masking the superiority of selective enamel etch when the whole restoration is viewed. However, the difference in the marginal integrity between both adhesive strategies was very low in both cases. For instance, the difference of the marginal integrity between selective enamel etch and self-etch was in no case larger than 3% regardless of whether the whole restoration or only enamel parts were considered. Furthermore, 3% ± 2.6% variability in the marginal integrity was reported when the marginal integrity of a restoration was evaluated by one calibrated operator at 2 different times.^29^ Therefore, it seems reasonable to assume that the observed statistical significant difference in the marginal integrity between selective enamel etch and self-etch (when considering only enamel) is indeed a “statistical detail” and would presumably not reflect a clinical significance.

A recent systematic review and meta-analysis reported that universal adhesives can be used in both adhesive strategies (self-etch and etch and rinse) in primary teeth. However, there was insufficient evidence about whether selective enamel etch is necessary when applying universal adhesives in primary teeth.^30^ In fact, this meta-analysis mentioned only one study that investigated the effect of selective enamel etch in combination with universal adhesives.^31^ This study concluded that enamel etching improves the bond strength of universal adhesives to primary enamel. However, the adhesion procedure in this study was conducted on flat ground enamel surfaces without preparing an actual cavity.^31^ Regardless, this study, and the meta-analysis, investigated the adhesive strategies that improve the bond strength to primary teeth, which might not directly correlate with the improvement of the marginal integrity investigated in the present study. In comparison to the recommended application time (20 seconds), the reduced adhesive application time (10 seconds) always caused a statistically significant decrease in the marginal integrity regardless of the viewed substrate. However, the difference in the marginal integrity between both application times was always equal or smaller than 8% with one exception (difference of 11%). Here again, it could be argued whether this difference would be of clinical relevance. Nevertheless, the observed marginal integrity at a 10-second application time frequently showed higher variance than the 20-second application time. Adhering to the recommended 20-second adhesive application time might therefore be more reliable.

To the authors’ best knowledge, no study has yet investigated the reduced adhesive application time in primary teeth. On bovine enamel, Karadas^32^ reported that the shortened application (5 seconds, no active moving) of 3 universal adhesives resulted in similar bonding durability as the manufacturer's recommended application time (10 to 20 seconds, with active moving). On human dentine, Maciel Pires et al^33^ reported that quick-and-passive application of universal adhesives had a jeopardising influence on the composite–dentine interface. At the microscopic level, the authors reported clear signs of phase separation and incomplete evaporation of the solvent with this kind of adhesive application. Another concern that was connected to the shortened adhesive application was the presence of completely free dentine tubules, which were not infiltrated with the adhesive.^33^ Also on human dentine, Saikaew et al^6^ reported that shortened application time (5 seconds, no active moving) of 3 universal adhesives negatively affected the bond strength. At the microscopic level, the authors also reported a phase separation with shortened application times. The authors also reported the formation of porosities, which represent entrapped solvents that failed to evaporate due to the shortened application time.^6^

Based on the present in-vitro study and within its limits, it can be concluded that universal adhesives offer comparable marginal integrity in class-II cavities in primary molars, regardless of the application mode (selective enamel etch or self-etch). Shortened application time (10 seconds) could lead to a reduction in the marginal integrity in comparison to the recommended application time of 20 seconds.

## Conflict of interest

Non disclosed.
